# **Associations of resilience with quality of life levels in adults experiencing homelessness and mental illness:** a **longitudinal study**

**DOI:** 10.1186/s12955-021-01713-z

**Published:** 2021-03-04

**Authors:** Cilia Mejia-Lancheros, Julia Woodhall-Melnik, Ri Wang, Stephen W. Hwang, Vicky Stergiopoulos, Anna Durbin

**Affiliations:** 1 MAP Centre for Urban Health Solutions, Li Ka Shing Knowledge Institute, St Michael’s Hospital, Unity Health Toronto, 30 Bonds Street, Toronto, ON M5B 1W8 Canada; 2 Department of Social Sciences, Faculty of Arts, University of New Brunswick, Saint John, Canada; 3 Division of General Internal Medicine, Department of Medicine, University of Toronto, Toronto, ON Canada; 4 Centre for Addiction and Mental Health, Toronto, ON Canada; 5 Department of Psychiatry, University of Toronto, Toronto, ON Canada

**Keywords:** Homelessness, Resilience, Quality of life, Substance use disorder, Mental illness

## Abstract

**Background:**

Homelessness constitutes a traumatic period that adversely impacts health and quality of life outcomes. The potential mitigating effects of resilience on quality of life levels in people experiencing homelessness are underresearched. This study assesses the longitudinal associations between resilience and quality of life scores among adults experiencing homelessness and mental illness.

**Methods:**

This study is a secondary analysis of longitudinal data collected over 6 years from participants (N = 575) of the At Home/Chez Soi study on Housing First, Toronto site. Repeatedly measured resilience scores are the primary exposure and repeatedly measured global quality of life scores and mental health-specific quality of life scores are the primary outcomes. Mixed effect models were used to assess the association between the exposures and the outcomes.

**Results:**

The majority of the participants were men (69.2%) and were on average 40.4 (± 11.8) years old at baseline. The average resilience score ranged between 5.00 to 5.62 over 8 data collection points across the 6-year follow-up period. After adjusting for gender, age, ethno-racial background, Housing First intervention, physical and mental comorbidities, and lifetime homelessness, higher resilience scores were positively associated with higher Global quality of life (Adjusted-coefficient: 0.23, 95% CI 0.19–0.27) and mental health-related quality of life values (Adjusted-coefficient: 4.15, 95% CI 3.35–4.95).

**Conclusion:**

In homeless adults with mental illness, higher resilience levels were positively associated with higher global and mental health related quality of life values. Further interventions and services aimed to enhance resilience mechanisms and strategies are warranted to enhance better mental health and quality of life outcomes of this population group.

***Trial registration*:**

At Home/Chez Soi trial was registered with ISRCTN, ISRCTN42520374. Registered 18 September 2009, http://www.isrctn.com/ISRCTN42520374.

## Background

Homelessness is a serious social and public health concern that affects thousands of people in both low-income and high-income countries [1]. It is a traumatic event that negatively affects health and well-being [2–6]. Those experiencing homelessness are often exposed to stressful conditions (e.g., lack of safety or a private space, perceived powerlessness and loss of social networks) commonly associated with shelter environments [2]. Additional traumatic and stressful experiences such as street victimisation, physical and sexual abuse, exposure to crime-related activities, and discrimination further compound the psychological effects of homelessness[4, 7, 8]. These traumatic events are often intertwined with trauma experienced prior to homelessness, such as adverse childhood experiences [9, 10], family dysfunction [11], or hazardous military service [12]. Exposure to trauma increases individual vulnerability to mental and substance use disorders, physical comorbidities, social disconnectedness, poor recovery outcomes and hopelessness [3, 4, 11, 13]. Indeed, traumatic experiences in this population are reflected in the high prevalence of post-traumatic stress disorder and other mental and substance use disorders [3, 4].

To overcome trauma, adversity, or stressful life changes, individuals have stress responses or adaptive mechanisms such as resilience [14–17]. Despite the lack of a universal definition of resilience, it is often referred to as biological, psychological and social processes or strategies that individuals have or adopt to resist or overcome life’s traumatic, stressful events and adversity [14–17]. Resilience is found to enhance individual adjustment or adaptation to new situations and allows people to bounce back from adversity or protect their mental health and wellbeing [14, 16–20]. In general, a high level of resilience has been found to improve mental health status, recovery (ability to function and live hopefully and meaningfully) and well-being outcomes [21, 22]. Similar positive effects of resilience are observed in adults affected by mental illness [18]. In socio-economically disadvantaged population groups, such as people experiencing homelessness, it has been found that more resilient individuals have higher levels of community functioning [23, 24] and social support [24], a higher percentage of days stably housed [24], and less suicidal ideation [25].

Despite their challenging social, housing and health conditions, some homeless people adopt resilience strategies to cope with their homeless state and life struggles. Some of these resilience strategies include use of affirmational statements, such as “stay strong and thankful,” “looking to live,” “hope to move forward,” “self-improvement,” “do not give up” [26], and remain “optimistic” and “confident” [27]. However, other less adaptive resilience strategies include negative feelings, emotional or psychological emptiness, pessimism and hopelessness [23, 26], which may hinder exits from homelessness and progress towards recovery. Resilience in homeless people is significantly negatively influenced by mental disorders (e.g., psychotic and depression disorders) [23, 28], which are frequently present in this population group.

Some researchers have studied the resilience process and outcomes in individuals experiencing homelessness, but there is still scant evidence on the association of resilience with overall and specific quality of life levels. Furthermore, the effects of resilience on well-being outcomes in homeless populations are often assessed using cross-sectional [23, 25] or qualitative methodological designs [26, 27]. To date, a handful of studies have explored this relationship over a short-term longitudinal period (e.g., ≤ 2 years)[24, 29]. The Toronto site of the At Home/Chez Soi (AH/CS) Housing First (HF) randomised trial collected six years of longitudinal data on quality of life and resilience measures among individuals with mental illness who were experiencing homelessness at the time of recruitment. The present study is a secondary analysis of these data. The objective of this study is to investigate the longitudinal associations of resilience levels with generic and mental health-related quality of life scores in adults who experience both homelessness and severe mental disorders.

## Methods

### Study population and design

The present study aimed analyses data from the Toronto site of the AH/CS study, which is part of the multi-site pragmatic randomised trial of HF in 5 cities across Canada (Toronto, Moncton, Montreal, Winnipeg and Vancouver) [30]. The Toronto AH/CS study design, population, tools and measures are published in detail elsewhere [31, 32]. Briefly, 575 participants were enrolled in the study between October 2009 and July 2011 and were followed up to March 2017 [32]. The primary participants’ inclusion criteria were as follows: (1) 18 years old or older; (2) homeless or precariously housed, with at least two episodes of absolute homelessness or one homelessness episode which lasted four or more weeks in the previous year; and (3) a diagnosed mental disorder with or without co-occurring substance or alcohol use disorder [31].

Participants were stratified according to their level of need for mental health services at the time of recruitment. High need participants were randomised to receive the HF intervention with assertive community treatment (ACT) and rent supplements or treatment as usual (TAU), which provided access to supportive housing and social and health services that were available in the community. Moderate need participants were randomised to either HF with intensive case management (ICM) plus rent supplements or to TAU. Detailed information on the specific level of needs criteria and the services provided for HF ACT, ICM treatment and TAU can be consulted in Hwang et al. [31].

The participants recruited for the Toronto AH/CS study site were initially followed for an average of 2 years (2009–2013) (phase 1) [32]. In 2014, they were re-enrolled in the study if willing to continue their participation (phase 2). A total of 414 of participants agreed to extend their participation for another two years and they were followed up to March 2017 [32]. The incompleteness of participants’ data over the follow-up period was due to participant attrition, missing interviews, missing item-responses, and low confidence in the participant questionnaire response assessed by the interviewer using an interviewer impression instrument [32].

### Ethics approvals

The AH/CS study of the Toronto site received ethics approval from the Research Ethics Board of St. Michael’s Hospital. At recruitment, all participants provided written informed consent to participate in the study. After an average of two years of follow-up, participants re-consented if they were willing to participate in the second phase of the study. The AH/CS study is registered with the International Standard Randomized Control Trial Number Register (ISRCTN42520374), http://www.isrctn.com/ISRCTN42520374

### Study measures

### Primary exposure

The overall resilience score, assessed using the abbreviate version of the Connor-Davidson Resilience Scale (CD-RISC2) [33, 34], is considered the primary exposure. The CD-RISC2, which included two items (“*Able to adapt to change,” “Tend to bounce back after illness or hardship”)* has been derived from the longer 25-item CD-RISC [33]. In previous studies, it has shown good internal consistency, test–retest reliability, and convergent and divergent validity, as well as high correlation with the overall 25-item CD-RISC score, being therefore, a good resilience indicator [34]. The CD-RISC2 was administered in face-to-face interviews at baseline, 12- and 24-months during phase 1 of the follow-up period, and at baseline, 6 and 18 months during the phase 2 of our study follow-up period. The Cronbach’s alpha coefficient of the CD-RISC in our study population was 0.92, which is indicative of great reliability. The overall resilience score was calculated by adding the scores (0–4) from the two items, which produced a total score with a range between 0 and 8. Higher values indicated more resilience.

### Quality of life outcomes

The main study outcomes are generic and mental health-specific quality of life scores. Generic quality of life was measured using the validated global 20-item of the Lehman’s 20-item QOL interview [35, 36], which measures QOL by assessing leisure, family and social relationships, finances, and safety domains. The abbreviated global 20-item was developed by Uttaro et al. using item-response theory and graded response modelling, with the purpose of reducing participant burden [36]. It retained similar internal consistency to that observed from the derived QOL dimensions scales of the Lehman’s 20-item QOL interview [36]. Therefore, it is a good single indictor to capture the overall essence of the subjective global quality of life. Further, it has previously used in study in homeless people [37] and in the context of the AH/CS study (data source of the present study) to assess changes in the QOL and the long-term effectiveness of HF on quality of life [32]. The Lehman’s 20-item QOL interview was administered in a face-to-face interview every six months during both phase 1 and phase 2 of the study follow-up [32]. The score for 20-item QOL ranges from 1 to 7-point order Likert scale, where the higher values indicate better overall quality of life.

Generic mental health related quality of life was assessed every six months only during phase 1 follow-up period using the EuroQol-5 vertical visual analogue scale (VAS) (0–100), which allows self-rated overall quality of life be linked to mental health status [38]. VAS values near 100 indicate high quality of life depending of their mental health. VAS format for measuring global QOL has been found to be valid, reliable and responsive as compared with QOL scores derived from other multi-item instruments[39], as it has showed moderate to high correlation with indicators of physical, psychological and social aspects of quality of life[39]. In our study population, assessing the mental health-related quality of life levels is critical as our participants had serious mental health disorders at baseline. Furthermore, previous studies carried out over phase 1 of the AH/CS study, find that participants continue to have high levels of severe mental health symptomatology over the two-years of follow-up [40–42]; therefore, it is likely that their mental health has continued to negatively affect their overall QOL over time. For the present study, the VAS was only analysed for phase 1 follow-up responses.

### Covariates

The following baseline characteristics were used as both adjusting factors and covariates. Age, categorized as < 30, 30–39, 40–49, ≥ 50 years, was used to capture potential effect differences between younger and older age groups. Gender was dichotomized to include the categories men and women, as only seven participants self-identified as transgender or transsexual, which is too small sample to preform meaningful analyses. Ethno-racial and cultural identity background was categorized as Aboriginal, black, white and other. Year of lifetime homelessness was categorized as < 3 and ≥ 3 years, where 3 years or more indicated homelessness chronicity[31]. Mental comorbidity was grouped as having < 3 or ≥ 3 mental disorders as an indicator of higher mental health comorbidity. All participants in the present study had at least one mental health concern. Similarly, we categorized physical comorbidity as having < 3 or ≥ 3 chronic diseases, as our participants had on average 2.0 of these health conditions. As our study participants were part of an HF randomised trial [31, 32], the intervention group HF vs. TAU was also included as an adjusting variable and covariate.

### Statistical analysis

The participants’ main characteristics were described (frequency and percentage) in the overall study sample. The resilience scores (primary exposure) and main outcomes (overall disease and mental health-specific quality of life scores) over the phase 1 and phase 2 follow-up periods were plotted by HF intervention group, as this study was embedded within a HF RCT.

The associations between resilience and the outcomes of interest were assessed using linear mixed effect models to account for repeated measures over a follow-up period of up to six years. Compound symmetry structure was used and all models were adjusted for age, gender, ethno-racial and cultural identity background, year of lifetime homelessness, mental comorbidity, physical comorbidity, and HF intervention group.

All analyses were tested at 0.05 statistical significance level. The “nlme package” in the R statistical software version 3.5.0 was used to perform the analyses for the present study.

## Results

The majority of study participants were men (68.36%). The mean age was 40.32 (SD 11.79) years, and majority of participants were from a black (34.55%) or white (35.27%) ethno-racial background (Table 1). 60.17% and 55.25% of participants had three or more mental disorders and physical chronic diseases, respectively. Slightly less than half of the participants (47.03%) had three or more years of lifetime duration of homelessness, and 52.35% received HF intervention, whereas 47.65% received TAU (Table 1).

**Table 1:** Description of the main charateristics of the study participants in the At Home/Chez Soi study, Toronto site

Main characteristics	All (N = 575)
*Age at enrolment in years*	
Mean (SD)	40.32 (11.79)
*Participant age groups (years)*	
< 30	138 (24.00%)
≥ 30–39	134 (23.30%)
≥ 40–49	182 (31.65%)
≥ 50	121 (21.04%)
*Gender*	
Men	376 (68.36%)
Women	167 (30.36%)
Other	7 (1.27%)
*Ethno-racial background*	
White	194 (35.27%)
Aboriginal	27 (4.91%)
Black	190 (34.55%)
Other	139 (25.27%)
*Total lifetime homelessness (years)*	
< 3	285 (52.97%)
≥ 3	253 (47.03%)
*HF intervention group*	
TAU	274 (47.65%)
HF	301 (52.35%)
*Number of psychiatric comorbidities*^a^	
< 3	229 (39.83%)
≥ 3	346 (60.17%)
*Number of physical comorbidities*^b^	
< 3	246 (44.73%)
≥ 3	304 (55.27%)

Current Major Depressive Episode, Current Manic Episode or Hypomanic Episode, Current PTSD, Current Panic Disorder, Current Mood Disorder with Psychotic Features, Current Psychotic Disorder, Current Alcohol Dependence, Current Substance Dependence, Current Alcohol Abuse, Current Substance Abuse, Current Suicidality

Asthma, stroke, Alzheimer's disease or dementia, Back problems, Dental problems, Foot problems,Skin problems, Arthritis An ulcer (stomach or intestine),Bowel problems (Crohn's disease or colitis), Chronic bronchitis or emphysema, Kidney or bladder trouble, urinary incontinence, High blood pressure, A thyroid condition, Heart disease, Diabetes, Liver disease (other than hepatitis), Cancer, Low iron anemia,tuberculosis, Hepatitis C, Hepatitis B, HIV/AIDS, Migraine headaches, Epilepsy or seizures

**Fig. 1:**
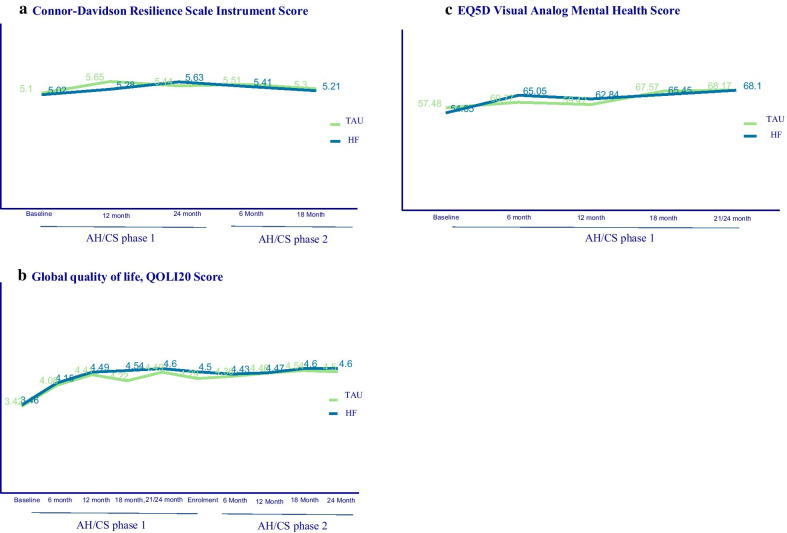
Distribution of resilience scores, global and mental health-specific quality of life scores over the phases 1 and 2 follow-up period, AH/CS study, Toronto site

The distribution of resilience scores, generic and mental health-specific quality of life scores over phases 1 and 2 of the follow-up period are presented according to HF treatment group in Fig. 1. The average scores for resilience slightly changed over the follow-up periods for HF and TAU participants, with values ranging between 5.00 to 5.21 over the six-year follow-up period. (Fig. 1a). The global quality of life scores ranged between 3.42 to 4.6 values over the six-year period (Fig. 1b), while the distribution of the mental-health quality of life scores over the two-year follow-up period ranged from 54.35 to 68.10 of the 100-maximum value (Fig. 1c).

The unadjusted and adjusted associations of resilience score with the quality of life outcomes are presented in Table 2. A one-point increase in resilience score was associated with higher values of global quality of life (Adjusted coefficient and 95% CI 0.23, 0.19–0.27) adjusting for gender, age, ethno-racial and cultural identity group, mental and physical comorbidities, lifetime duration of homelessness, and HF treatment group. A positive association between resilience levels and mental health-specific quality of life level was also observed, where one increased point in the resilience score was associated with an increase of 4.15 (95% CI: 3.35 to 4.95) points in participants’ mental health-related quality of life after adjusting for sociodemographic characteristics, health related comorbidities and HF intervention group (Table 2.)

**Table 2:** Unadjuted and adjusted associations of resilience scores with global disease-quality of life scores and mental health-specific quality of life score in particpants of the At Home/Chez Soi participants, Toronto site

Models		Global quality of life (Global 20-item QOL score, range 1–7)			Mental health-specific quality of life (EuroQol-5 mental health-VAS score, range 0–100)			
		Coefficient	95% CI	*P* value		Coefficient	95% CI	*P* value
*Unadjusted model*		0.23	0.19; 0.28	< 0.001				
Observations	N = 2015				N = 1105			
*Resilience scale score*	Per increase of 1 (range: 0–8	− 0.07	− 0.11; − 0.03			4.31	3.51; 5.10	< 0.001
*Adjusted model*								
Observations	N = 1985				N = 1088			
*Resilience scale score*	Per increase of 1 (range: 0–8)	0.23	0.19; 0.27	< 0.001		4.15	3.35; 4.95	< 0.001
Self-Identified gender	Women vs. Men	− 0.04	− 0.27; 0.19	0.74		− 0.85	− 4.53; 2.83	0.65
Participant age group at baseline	30–39 vs. < 30	0.20	− 0.12; 0.51	0.22		− 2.15	− 7.14; 2.84	0.40
	40–49 vs. < 30	− 0.02	− 0.31; 0.27	0.89		− 0.85	− 5.49; 3.79	0.72
	50 + vs. < 30	0.07	− 0.26; 0.39	0.69		0.36	− 4.91; 5.64	0.89
Ethno-racial groups	Aboriginal vs. White	0.63	0.13; 1.13	0.01		− 0.62	− 8.55; 7.31	0.88
	Black vs. White	0.12	− 0.14; 0.38	0.36		2.78	− 1.33; 6.89	0.18
	Other vs. White	0.02	− 0.26; 0.30	0.88		− 1.70	− 6.17; 2.78	0.46
HF intervention group	HF vs. TAU	0.17	− 0.04; 0.38	0.11		1.85	− 1.52; 5.23	0.28
Number of physical comorbidities	≥ 3 vs. < 3	− 0.43	− 0.66; − 0.20	< 0.001		− 7.48	− 11.24; − 3.73	< 0.001
Number of psychiatric comorbidities	≥ 3 vs. < 3	− 0.48	− 0.71; − 0.25	< 0.001		− 6.47	− 10.23; − 2.72	< 0.001
Total lifetime homelessness(years)	≥ 3 vs. < 3	0.02	− 0.20; 0.24	0.87		2.64	− 0.93; 6.22	0.15

## Discussion

This longitudinal study of adults experiencing homelessness and mental illness identified that high levels of resilience were positively associated with greater global and mental quality of life scores. Among these associations, higher values were observed for the mental health-related quality of life, where one increased point in the resilience score was associated with an increase of 4.15 points in the mental health-specific quality of life score.

Resilience is individual quality that allows coping with stressful life experiences [14–17], including adversity surrounding homelessness [23, 26–28, 43] and mental illness [18]. In this study, participants’ resilience scores over the six-year follow-up period ranged between 5.01 to 5.63 points, over the 0–8 range of values. These scores are similar to those observed in non-homeless populations outside Northern American settings, using the same 2-item resilience scale [44, 45], but lower compared to scores in the general population within the US context, where resilience levels average 6.91 points [34]. This finding suggests that people with experiences of homelessness and serious mental disorders can leverage strategies to strengthen resilience and adversity while experiencing unstable housing.

Among the strategies that people leverage to overcome homelessness and its sequelae, some may promote their health and well-being. For example, individuals may seek instrumental support, socialize, engage in meaningful activities, and maintain hopefulness [26, 27, 46]. However, other strategies or behaviours used as adaptive mechanisms may have a negative impact on health and other outcomes. Among those maladaptive strategies are drug and alcohol use [27, 47] and engagement with criminal-related activities[48]. Thus, it is crucial to facilitate access to and provide social, psychological, emotional, and health support and services to boost this population’s resilience and to enhance other life dimensions, such as health, mental health, and quality of life. Our findings offer some promising insights in this little studied or understood area.

In our study population, we found that people with higher scores of resilience also had better global quality of life levels over the six-year follow-up period, as well as higher mental health-related quality of life values over the first two-years of follow-up. In the general population, quality of life and substance use recovery are influenced by a wide range of multidimensional factors[49]. These factors become more varied and complex in the context of housing instability, homelessness, and mental illness [37, 50–54]. Resilience is considered an important life and health protective factor [55], as it can help homeless people look forward to life in a hopeful way even when they face adversity and social barriers. Further, resilience can facilitate integration in communities, build family and social relationships and networks, and allow for participation in meaningful activities (e.g., work, spirituality, leisure, training-related activities) [23, 27]. All of this, in turn, could lead to improvements in quality of life for persons experiencing homelessness and mental health concerns.

A study carried out among 410 Dutch homeless persons [55] found that most of participants have at least one personal life goal for their near future, and among these goals, building resilience were among those aspects that participants’ were looking forward to [55]. It also found that higher levels of goal-related self-efficacy were positively associated with higher quality of life values [55]. Another study conducted with youth who experience homelessness used latent class analysis, found that a greater level of resilience acts as a protective factor which is associated with improved quality of life [29]. The existing evidence and findings from the present study support the positive and instrumental role of resilience in achieving greater quality of life levels among people with experiences of homelessness and mental illness.

The present study has some limitations. Resilience and the studied outcomes were measured contemporaneously over the follow-up period; therefore, we cannot exclude potential reverse associations. Mental health-related quality of life was only analysed in phase 1 of the AH/CS study; hence, the observed findings may have differed if this measure had been analysed over the entire six-year follow-up period. Further, using the VAS for assessing the mental health-related quality of life may only capture the effect of a specific mental health disorder (e.g., depression) rather than the effects of all aspects of mental health status, which influence quality of life [39]. It is also likely that due to potential physical, cognitive and mental impairment of some participants, the VAS rating could be susceptible to marking error; therefore, VAS scores may not be reflective of actual mental health quality of life levels and potential changes over time. Despite these limitations, existing evidence indicates that the VAS is a valid instrument to capture respondents’ perspectives on their quality of life [56]. It has good inter-rater reliability and test–retest reliability when compared with quality of life scores derived for multi-dimensional or multi-item instruments [39]. In the present study, measures of objective quality of life were not included. Therefore, the associations between resilience and specific objective quality of life indicators (e.g. income) may differ from those observed in the present paper, which assesses subjective quality of life. Yet, if a person perceived their quality of life as poor, it is going to negatively affect overall well-being, even if an objective tool states that they have the resources for a high quality of life. Further, this study assesses general and mental health related quality of life, rather than focusing on specific indicators (e.g., employment, social networks) or dimensions level; therefore, the relationship between individual quality of life indicators and resilience should be explored in future studies. Finally, the present study was embedded in a pragmatic randomised trial with people experiencing both homelessness and mental illness. Thus, the findings may not be generalisable to all homeless individuals or to other settings.

The present study has implications for practice and policy. Our findings revealed that people experiencing both homelessness and mental illness have moderate resilience levels, and these are positively associated with improvements in their global and mental health-related quality of life levels over time. Therefore, there is an opportunity to implement interventions to further enhance resilience and coping strategies that may have lasting impacts on mental health, substance use and quality of life. Finally, social and health providers working with homeless people could also incorporate resilience-based psychoeducation services [19], where specific skills and capabilities such as active problem-solving, cognitive reappraisal, guided-self-dialogue, social support and competence building, learned optimism, and stress management could be enhanced to help this population group grow their resilience and overcome trauma and distress [15, 19].

In conclusion, higher resilience levels are positively associated with higher long-term global and mental-health related quality of life values in homeless adults with mental illness. Further interventions and services aimed to enhance resilience mechanisms and strategies are warranted to enhance better mental health and quality of life outcomes of this population group.

## Data Availability

The At Home/Chez Soi study dataset cannot be made publicly available due to the sensitive nature of the data and agreements and procedures governing the use of the dataset that were established by the study sponsor, the Mental Health Commission of Canada. However, anonymized participant data from the AH/CS study, as well as the specific dataset used in the present paper, can be made available to investigators who complete the following steps: (1) present a study proposal that has received approval from an independent research committee or research ethics board; (2) provide a data request for review by the AH/CS data access committee; (3) following approval of the request, execute a data-sharing agreement between the investigators and the AH/CS data custodians. Study proposals and data access requests should be sent to Evie Gogosis (Evie.Gogosis@unityhealth.to), research manager for the Toronto site of the AH/CS study, and to Dr. Stephen Hwang (Stephen.Hwang@unityhealth.to), co-principal investigator of the Toronto site of the AH/CS study.

## Abbreviations

AH/CS: At Home/Chez Soi
ACT: Assertive community treatment
CD-RISC: Connor-Davidson Resilience Scale
CI: Confidence interval
HF: Housing first
ICM: Intensive case management
QOL: Quality of life
RCT: Randomized controlled trial
TAU: Treatment as usual
VAS: Vertical visual analogue scale

## Code availability

The statistical code used in this study could be required to the corresponding author. The request will be revised by the AH/CS research team before it can made available.
